# Incidence, risk factors, and burden of incisional hernia repair after abdominal surgery in France: a nationwide study

**DOI:** 10.1007/s10029-023-02825-9

**Published:** 2023-06-27

**Authors:** P. Ortega-Deballon, Y. Renard, J. de Launay, T. Lafon, Q. Roset, G. Passot

**Affiliations:** 1 Service de Chirurgie Générale, Digestive, Cancérologique et Urgences, CHU de Dijon - CR INSERM 1231 – CIC 1432, Module Épidémiologie Clinique – Université de Bourgogne, 14, rue Paul Gaffarel, 21079 Dijon Cedex, France; 2 Service de Chirurgie Générale, Digestive et Endocrinienne, CHU de Reims, Reims, France; 3 Department of Medical Affairs, Becton, Dickinson and Company, 11 Rue Aristide Berges, 38800 Le Pont-de-Claix, France; 4 Heva, 186 avenue thiers, 69600 Lyon, France; 5 Service de Chirurgie Digestive et Oncologique, Hôpital Lyon Sud, Hospices Civils de Lyon, 165 Chemin du Grand Revoyet, 69310 Pierre-Bénite, France

**Keywords:** Incisional hernia repair, Laparotomy closure, Cost, SNDS

## Abstract

**Purpose:**

Incisional hernias are common after laparotomies. The aims of this study were to assess the rate of incisional hernia repair after abdominal surgery, recurrence rate, hospital costs, and risk factors, in France.

**Methods:**

This national, retrospective, longitudinal, observational study was based on the exhaustive hospital discharge database (PMSI). All adult patients (≥ 18 years old) hospitalised for an abdominal surgical procedure between 01-01-2013 and 31-12-2014 and hospitalised for incisional hernia repair within five years were included. Descriptive analyses and cost analyses from the National Health Insurance (NHI) viewpoint (hospital care for the hernia repair) were performed. To identify risk factors for hernia repair a multivariable Cox model and a machine learning analysis were performed.

**Results:**

In 2013–2014, 710074 patients underwent abdominal surgery, of which 32633 (4.6%) and 5117 (0.7%) had ≥ 1 and ≥ 2 incisional hernia repair(s) within five years, respectively. Mean hospital costs amounted to €4153/hernia repair, representing nearly €67.7 million/year. Some surgical sites exposed patients at high risk of incisional hernia repair: colon and rectum (hazard ratio [HR] 1.2), and other sites on the small bowel and the peritoneum (HR 1.4). Laparotomy procedure and being ≥ 40 years old put patients at high risk of incisional hernia repair even when operated on low-risk sites such as stomach, duodenum, and hepatobiliary.

**Conclusion:**

The burden of incisional hernia repair is high and most patients are at risk either due to age ≥ 40 or the surgery site. New approaches to prevent the onset of incisional hernia are warranted.

**Supplementary Information:**

The online version contains supplementary material available at 10.1007/s10029-023-02825-9.

## Introduction

Laparotomies are frequent operations, either for elective procedures or for urgent surgery. In France, in 2010, they amounted to 430000 procedures [1]. Incisional hernia is a common complication of laparotomies [2] and may have been considered as a risk worth taking for a life-saving surgical procedure [3, 4]. While probably underreported, incisional hernia is estimated to develop in 4–15% of patients after laparotomy [1, 5, 6], or 3–4% within 4–5 years of abdominal surgery [7, 8]. The burden of incisional hernia encompasses substantial morbidity [9], impaired quality of life [10], possible hospital admissions for a surgical procedure, and leads to expenses for the health insurance fund [8, 11, 12]. As evidence, in 2011, based on a sample of 51 French public hospitals, the mean hospital cost of incisional ventral hernia repair was €4731 [13].

Previous studies have identified risk factors in small populations in other countries [7, 9, 14–16] and in France [1]. Identifying risk factors of incisional hernia repair in France and at national level with up to date data could enable the selection of higher-risk patients indicated for specific abdominal prophylactic surgical procedures or for enhanced follow-up to ultimately decrease the burden of incisional hernia repair. Likewise, the economic burden of incisional hernia has been examined in other populations [8, 11, 12] but not at national level in France.

This national study examined incisional hernia repair (i.e., any patient requiring a surgical procedure at the hospital) within 5 years of abdominal surgery in France. Our aims were to assess the public health burden of incisional hernia repair (incidence, recurrence, and hospital-related costs) and to identify the risk factors of incisional hernia repair.

## Methods

### Study design and data source

A national, retrospective, longitudinal, observational study including all French hospitals was carried out. The study was based on the exhaustive hospital discharge database (PMSI, Programme de Médicalisation des Systèmes d’Information) of the French National Health Insurance (NHI) database (SNDS, Système National des Données de Santé) [17]. The PMSI database covers all stays in medical, surgical, or obstetric facilities in all public and private hospitals. It includes all medical and surgical procedures undertaken during inpatient stays or outpatient visits. All procedures are coded with the Common Classification of Medical Procedures (CCAM) —the French equivalent of the American Current Procedural Terminology (CPT). In addition, the reasons for hospitalisation and the patient’s medical history are documented using one or more ICD-10 codes (as principal diagnosis, PD, related diagnosis, RD, and significant associated diagnosis, SAD).

### Study population and study period

All adult patients (≥ 18 years old) hospitalised for an abdominal surgical procedure (“index surgery” performed during the “index hospital stay”) (list of procedures in Supplement Table 1) during the inclusion period (between 01-01-2013 and 31-12-2014) and later admitted to the hospital at least once for an incisional hernia repair within 5 years of the index surgery were included. Hernia ICD-10 code K432 (Incisional hernia without obstruction or gangrene) and/or incisional hernia repair CCAM codes LMMA004, LMMC015, LMMA010 were sought on the hospital discharge summary, as the PD, a RD, or a SAD. The description of the burden of incisional hernia repair was based on this study population.

To make sure that the incisional hernia repair was the consequence of the index surgery, patients with the following confounding biases were excluded: 1/those with another digestive surgery procedure performed within the two years prior to the index date; or 2/those with a previous incisional hernia repair within the two years prior to the index date; or 3/those with a PD, RD, or SAD of digestive or urinary stoma during a hospital stay within the two years prior to the index date (codes used to identify the stoma in Supplement Table 2); or 4/those with an abdominal surgery procedure between the index date and the first hospital stay for an incisional hernia repair. This step increased the likelihood of an association between the index surgery and the incisional hernia repair, at the cost of a reduced number of patients included in the Cox model analysis. For the machine learning analysis, the selected population was further restricted to patients with five years of follow-up.

Patients presenting with incisional hernia at the time of index surgery were excluded, as well as patients with a history of incisional hernia. The goal was to study a population totally free from any incisional hernia before inclusion.

When a study patient underwent several surgical procedures at different anatomic sites during the index hospital stay, the surgical procedure potentially responsible for the hernia was determined following an algorithm detailed in Supplement Methods 1.

The index date was defined as the date of the first abdominal surgery during the inclusion period. A historical period of 2 years prior to the index date was considered to capture the patient’s medical history. All patients were followed for 5 years from the index surgery, until the end of the study (31-12-2019), or death, whichever occurred first.

As the PMSI database exhaustively reports all hospital stays in France, all patients in the PMSI meeting the selection criteria were included and no computation of the study size was performed.

### Outcome measures

For each patient, information was documented at the time of the index hospitalisation on age, sex, field of surgery (Supplement Table 1). “Peritoneal procedures” were all procedures only affecting the peritoneum, without any organ resection. The presence of important chronic comorbidities (Supplement Table 3) identified as PD, RD or SAD in the patient discharge summary were sought for the index hospital stay as well as all hospital stays during the two-year follow-back period.

For both the index hospital stay and the incisional hernia repair hospital stays, the following variables were documented as follows: length of stay, hospital status (private or public), annual number of abdominal surgeries of interest (in quartiles), presence of post-operation infection (ICD-10 code T814 as PD, RD, or SAD) and time between surgery and infection, referral to the hospital through the emergency room, and cost. In addition, the time between the index hospital stay and the first incisional hernia repair hospital stay was computed, and the volume of activity of the hospital for the incisional hernia hospital stay (the annual number of abdominal surgeries of interest performed in that hospital, in quartiles).

Obesity was captured with ICD-10 code E66 on the patient discharge form.

### Statistical methods

Continuous data were summarized by their mean, standard deviation (SD), median, and first (Q1) and third (Q3) quartiles. Categorical data were summarized by percentage.

### Cost analyses

The costs are presented from the point of view of the French NHI and were based on national costs applicable from 2013 to 2019. Costs are in Euros of the corresponding year. The official flat fee of the hospital stay was applied according to the DRG (Diagnosis Related Group or GHM, Groupe Homogène de Malades) code attributed in the PMSI database. These standard tariffs include medical and related procedures, nursing care, treatments (except specific expensive drugs and medical devices), hospitality, and related investment costs for hospitalised patients. Additional costs per day of hospitalisation in an intensive care unit were added where appropriate. For private hospitals, where physicians are paid on a fee-for-service basis, physician fees were identified from the French observatory of real-world spending on healthcare (ENCC, Etude Nationale des Coûts à méthodologie Commune) [18] and added to the private DRG tariff. Expensive drugs and implantable medical devices were costed using the retail price listed in the official public database named FICHCOMP.

Given that all healthcare consumptions are reported in the database, no replacement of missing values were required.

### Analysis of risk factors for incisional hernia repair

To provide an estimation of the association of risk factors and the occurrence of incisional hernia repair, a Cox regression model was implemented, and the results were presented as hazard ratios (HR) and their 95% confidence intervals (CI). First, a univariate analysis was performed. Then, only the factors with a *p*-value ≤ 0.05 were retained for a multivariable analysis. The models were adjusted on all variables.

To identify patients at higher risk of incisional hernia repair within 5 years of the index surgery, a machine learning model was built. The model used a binary splitting decision tree algorithm [19, 20] which allows to extract rules from the outcome profiles [21]. The list of candidate variables to best discriminate the study population according to the presence or absence of an incisional hernia repair with 5 years listed in Supplement Table 4.

The model was initially constructed on a random sample of 80% of the study population (the training set). The model was built iteratively, starting with the full training set.

First, the model searched for the most discriminating variable to divide the population into two groups (presence vs. absence of at least one hospitalized incisional hernia within 5 years). The quality of splits was estimated by the Gini impurity statistic. The higher the statistic, the most discriminating the split.

In each following round, each group was split into two subgroups using the same procedure, thereby creating a binary tree. Further subgroups were developed until any of the folloewing three criteria were met:

- The resulting subgroup corresponded to < 1% of the original cohort,
- The gain in Gini impurity statistic was minimal (< 10^–7^)
- Six levels of division from the initial population had been reached.

Second, the model’s performance was evaluated on the remaining 20% of the study population (the test set) to validate the model and to control for overfitting. The model was considered validated if the decision tree hierarchy in the test set matched that in the training set and if the performance (accuracy, recall, precision and f1 score) in the two sets were high and comparable.

Finally, if the model was considered validated in the validation set, then the model was frozen. The two sets were combined, and the model reiterated on the complete study population to generate the results.

To prevent censorship-related bias, all patients with less than 5 years of follow-up and no hernia were excluded from the analysis. The results were presented as relative risks (RR) with their 95% CI. The machine learning analysis was independently performed for each field of surgery, constituting the first level of the patient profiles. The subsequent levels were determined by the machine learning, i.e., the algorithm process was unsupervised.

All applicable statistical tests were two-sided and were performed at a 5% significance level. All patients meeting the selection criteria were included; no computation of the sample size was carried out.

Statistical analyses were performed using SAS version 9.4.

## Results

### Study populations

**Fig. 1:**
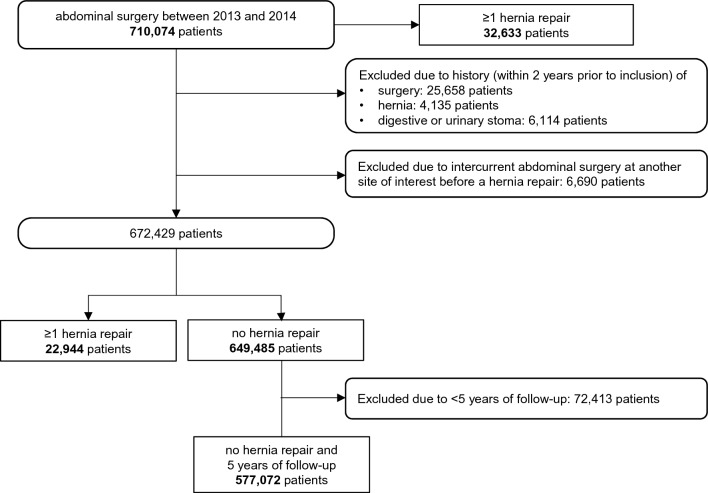
Patient selection process

Between 2013 and 2014, 710074 patients underwent abdominal surgery. The mean follow-up duration was 4.6 years (± 1.2, Q1–Q3 5–5 years), corresponding to a total of 3.3 million patient-years. Among them, 32633 patients (4.6%) had at least one incisional hernia repair within 5 years of the index surgery and 677441 patients (95.4%) had no incisional hernia repair (Fig. 1).

For the statistical analyses, after applying the exclusion criteria to increase the likelihood of a relationship between the index surgery and the incisional hernia repair, 672429 patients were considered (94.7% of 710074 patients). Among them, 22944 patients (3.4% of 672429) had at least 5 years of follow-up and underwent at least one incisional hernia repair within 5 years of the index surgery. For the Cox model, these 22944 patients were compared to the 649 485 patients without incisional hernia repair within 5 years (regardless of their follow-up duration) and for the machine learning analysis, to 577072 patients without incisional hernia repair and alive after 5 years of follow-up.

### Characteristics of patients with an abdominal surgery and those with an incisional hernia repair within 5 years, along with their index hospital stays

At their index hospital stay, the 32633 patients with at least one incisional hernia repair were older than the whole population of the 710074 patients with an abdominal surgery (on average, 59.6 years old vs. 53.6 years old) and more of them were male (14680 or 45.0% vs. 268339 or 37.8%) (Table 1). Colorectal and hepatobiliary surgeries were the most common fields of index surgery in both populations, but the proportion of colorectal surgery was much higher in patients with at least one incisional hernia repair than in the whole population of patients with abdominal surgery (15876 patients or 48.6% vs. 234611 or 33.0%). All comorbidities reported during the index hospital stay were more common in patients with subsequent hernia repair, especially cancer (10835 patients, 33.2%) and obesity (9565 patients, 29.3%) and their mean index hospital stay duration was 12.6 (± 14.5), compared to 8.0 days (± 12.3) for all patients with abdominal surgery.

**Table 1:** Characteristics of patients with an abdominal surgery, patients with an incisional hernia repair within five years, and their index hospital stay

Characteristic	Patients with abdominal surgery in 2013–2014 *N* = 710,074	Patients with incisional hernia repair within 5 years *n* = 32,633
Age (years), mean (± SD)	53.6 (± 19.3)	59.6 (± 14.7)
Median, Q1–Q3	54, 38–68	61, 49–70
Sex, No. (%) male	268339 (37.8%)	14680 (45.0%)
Field of surgery, No. (%)		
Hepatobiliary	250763 (35.3%)	6225 (19.1%)
Colon and rectum	234611 (33.0%)	15 876 (48.6%)
Stomach, duodenum	112024 (15.8%)	4583 (14.0%)
Other procedures on the small bowel or the peritoneum	105272 (14.8%)	5422 (16.6%)
Pancreas	7404 (1.0%)	527 (1.6%)
Medical history, No. (%)		
Obesity	161178 (22.7%)	9565 (29.3%)
Cancer	147300 (20.7%)	10835 (33.2%)
Diabetes	72253 (10.2%)	4620 (14.2%)
COPD	48721 (6.9%)	3621 (11.1%)
Heart failure	21488 (3.0%)	971 (3.0%)
Renal failure	20959 (3.0%)	1185 (3.6%)
Cirrhosis	5548 (0.8%)	410 (1.3%)
Index hospital stay duration (days) mean ± SD	8.0 ± 12.3	12.6 ± 14.5
Median, Q1–Q3	4, 2–9	9, 5–15
Index hospital status, No. (%)		
Public	398877 (56.2%)	18164 (55.7%)
Private	311197 (43.8%)	14469 (44.3%)

Q1–Q3 first and third quartiles; *SD* standard deviation

Among the patients with at least one incisional hernia repair, 9.3% (3029 patients), 3.9% (1275 patients) and 2.5% (817 patients) suffered from an infection during the index hospital stay, after the index hospital stay but within 30 days of the index hospital stay, and between 31 and 90 days after the index hospital stay, respectively.

Among the 32633 patients with at least one incisional hernia repair, 27516 patients (84.3%) had one hernia repair and 5117 patients had at least two hernia repairs, corresponding to a recurrence rate of 15.6% (distribution of incisional hernia repairs per patient in Supplement Fig. 1).

### Characteristics and cost of the incisional hernia repair hospital stay 

The mean duration of the hospital stay for the incisional hernia repair of the 32633 patients was 6.3 days (± 8.6) (Table 2). The mean time between the index surgery and the hernia repair was 22.1 months (± 15.2) (i.e., 1.8 years ± 1.3) and the median time was 17.8 months. Six percent of patients were referred to the hospital for their hernia repair from the emergency room.

**Table 2:** Incisional hernia repair hospital stay characteristics and cost

Characteristic	Patients with incisional hernia repair within 5 years *n* = 32 633
Hospital length of stay (days) mean ± SD	6.3 ± 8.6
Median, Q1–Q3	5, 2–7
Hospital status, No. (%)	
Public	18116 (55.5%)
Private	14517 (44.5%)
Time between index surgery and first hernia repair (months)	
Mean ± SD	22.1 ± 15.2
Median, Q1–Q3	17.8, 9.8–32.2
Referral from emergency room (yes), No. (%)	1967 (6.0%)
Cost (€), Mean (± SD)	4152.5 (± 5210.0)
Median, Q1–Q3	3007.4, 2004.6–4137.2

Q1–Q3 first and third quartiles; *SD* standard deviation

The mean cost of the hospital stay covered by the NHI for an incisional hernia repair was reported to be €4153 (± €5210). Consequently, the cumulative hospital cost incurred by all incisional hernia repairs considered in our study was €135.5 million. In other words, for all patients undergoing abdominal surgery in a given year, the NHI pays €67.7 million over five years for subsequent incisional hernia repairs.

### Risk factors of hernia repair after abdominal surgery

#### Cox multivariable analysis

**Fig. 2:**
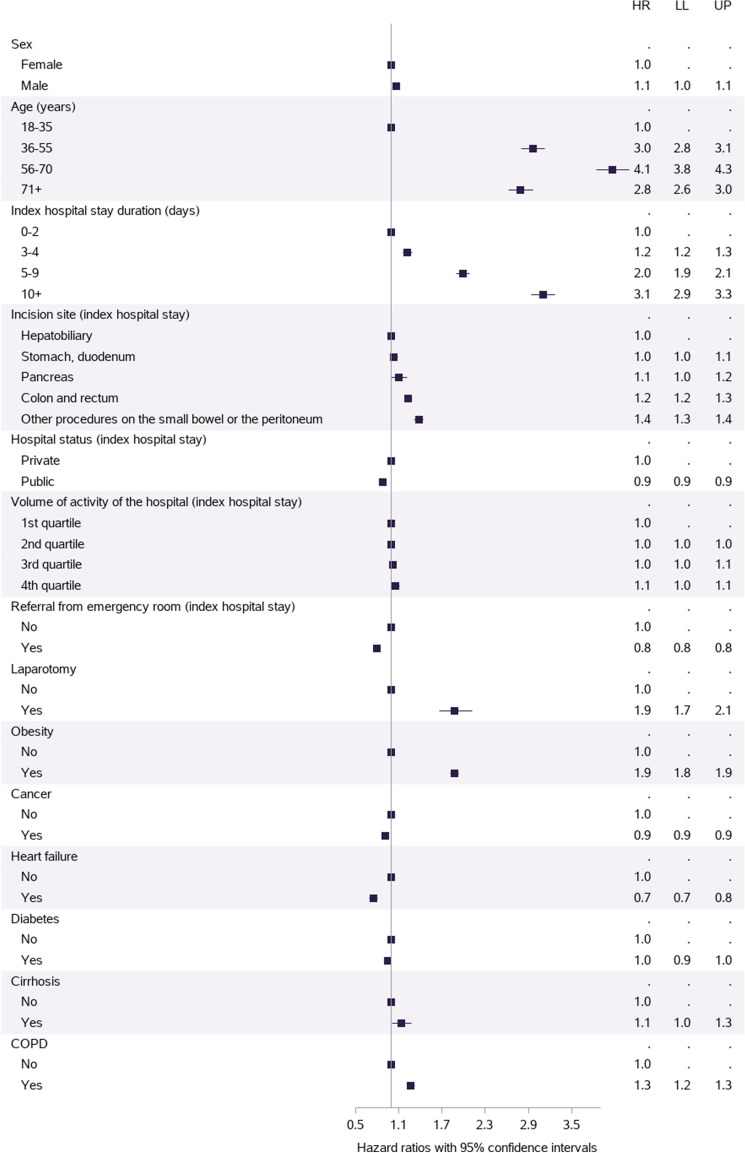
Multivariable analysis of factors associated with the first hospitalised incisional hernia. Volume of activity of the hospital for the incisional hernia hospital stay (the annual number of abdominal surgeries of interest performed in that hospital, in quartiles). *HR* hazard ratio; *LL* lower limit of the 95% confidence internal; *UP* upper limit of the 95% confidence interval; *COPD* chronic obstructive pulmonary disease. Undergoing a laparoscopy or chronic kidney disease was not associated with incisional hernia repair (not shown on the figure)

Several factors were significantly associated with undergoing a hernia repair within 5 years of abdominal surgery (Fig. 2, Supplement Table 5). After adjustment, the older the patient, or the longer the index hospital stay, the higher the risk of hernia repair (HR 4.1 [95% CI 3.8 to 4.3] for patients aged 56–70 and HR 3.1 [2.9 to 3.3] for hospital stay ≥ 10 days). The risk of hernia repair nearly doubled after having a laparotomy (compared to a laparoscopy) (HR 1.9 [1.7 to 2.1]) or when being obese (vs not obese) (HR 1.9 [1.8 to 1.9]). There was a small but significant added risk for male patients (HR 1.1 [1.0 to 1.1]). Compared to having an index hepatobiliary surgery, the risks were 1.4 times higher [1.3 to 1.4] for procedures on the small bowel or the peritoneum and 1.2 times higher [1.2 to 1.3] for colorectal surgery. Finally, the HR for suffering from COPD (vs not suffering from COPD) was 1.3 [1.2 to 1.3].

#### Machine learning

The machine learning analysis was carried out on 600016 patients: 577072 patients with at least 5 years of follow-up and no hernia, and 22944 patients with at least one hernia repair (regardless of their follow-up duration) (Fig. 1).

**Fig. 3:**
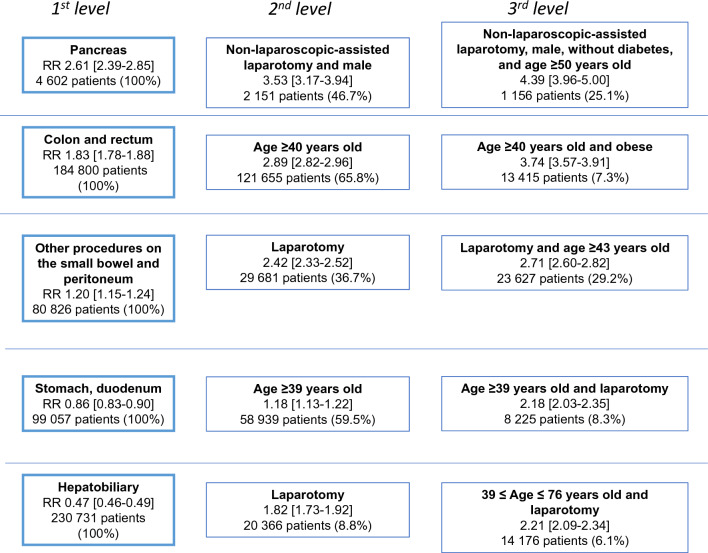
Factors associated with incisional hernia repair according to the machine learning analyses. For each field of surgery, the machine learning approach iteratively determined the strongest risk factors for incisional hernia repair and quantified the risk with relative risks (RR)

The onset of an incisional hernia repair within five years was related to the field of surgery. The field of surgery was, therefore, chosen as the first level of the patients’ profiles (Fig. 3). Compared to all patients with abdominal surgery (any field of surgery), the procedures with the highest risks of subsequent incisional hernia repair affected the pancreas (RR 2.61 [95% CI 2.39 to 2.85]) and the colon and rectum (RR 1.83 [1.78 to 1.88]).

For each field of index surgery, the profile of the patients with the highest risk varied, but age above 40 years and laparotomy appeared to be common risk factors. For instance, the risk of hernia repair after colorectal surgery was higher in patients aged 40 and above (RR 2.89 [2.82 to 2.96]) and even higher (RR 3.74 [3.57 to 3.91]) in patients aged 40 and above and obese. Even among patients operated on fields of surgery with lower risk of hernia repair (stomach, duodenum RR 0.86 [0.83 to 0.90] and hepatobiliary RR 0.47 [0.46 to 0.49]), being 39 years old or above (for stomach, duodenum) and an index surgery performed by laparotomy (both fields of surgery) increased the risk of hernia. Additional risk profiles (including obese patients) carried by a smaller proportion of patients are shown in Supplement Fig. 2.

## Discussion

In this French study on all 710074 patients hospitalised in 2013–2014 for abdominal surgery, 4.6% of them (32633 patients) required an incisional hernia repair within five years of the surgical procedure and half of the hernia repairs occurred within 18 months. The recurrence rate after repair was 15.6% (5117 patients). Each incisional hernia repair costs €4153 in hospital care to the NHI. The Cox model and the machine learning unveiled similar risk factors. Some fields of digestive surgery put patients at high risk of incisional hernia: colorectal, and small bowel and peritoneum. Age > 40 also increased this risk. The Cox model identified other risk factors such as obesity and longer hospital stays.

The Cox analysis identified pancreatic, colorectal, small bowel and peritoneal procedures as those carrying the highest risk for incisional hernia repair. The machine learning approach excluded patients with incomplete follow-up, thereby excluding patients who died before the end of the study. Patients with pancreatic surgery have a very low survival rate (11% five years after diagnosis for pancreatic cancer patients [22]). The machine learning approach compared the patients who survived at least 5 years and had no incisional hernia repair (i.e., excluded patients who died within 5 years without hernia) to the patients with incisional hernia repair, regardless of the follow-up duration. Therefore, the RR for pancreatic cancer is likely overestimated by the machine learning. In contrast, the Cox analysis included deceased patients (removing the survival selection bias) and showed no added risk for pancreas surgery patients. The machine learning analysis confirmed the risk factors identified with the Cox analysis (namely age above 35 years old, laparotomy, and obesity) and added the possibility of identifying high-risk patient profiles by combining risk factors. As a result, age above 40 appeared to be a key risk factor, even for low-risk procedures. It also identified hepatobiliary surgery as a lower risk operation compared to others, probably due to the high presence of laparoscopic cholecystectomies. However, hepatobiliary surgery performed by laparotomy almost doubled the risk of incisional hernia. In a US study, incisional hernia prevailed in colorectal (10%) and hepatobiliary (8%) procedures and obesity, age, open surgery, and prior surgery were independent risk factors across surgical specialties [8].

Finally, the high recurrence rate of incisional hernia repair, already shown in previous studies [8], demonstrated that the procedure was not a definitive solution to hernia and that it was a stand-alone risk factor. Patients may enter a vicious circle of incisional hernia repairs [23]. Prevention of incisional hernia is hence particularly warranted, including by considering incisional hernia and hernia recurrence risk factors.

In France, an earlier study on incisional hernia repair on the same database but restricted to patients who underwent a laparotomy (including for cardiovascular procedures) found that 5% of patients had an incisional hernia repair within years of the laparotomy [1]. The main individual risk factors for incisional hernia repair were diabetes, high blood pressure, and, consistent with our findings, COPD, obesity, and older age (as early as 39 years old). As more surgical procedures are performed using the laparoscopic technique [12], the incidence of hernia repair should decrease [4]. In 2011, in a study on 3239 patients in a sample of 51 French hospital estimated the hospital-related incisional ventral hernia repair to be €4731 [13], close to our finding of €4153. This study further assessed the mean indirect cost (sick leave) of incisional hernia repair in working patients to be €5376.

This study has several strengths. First, it is based on real-life data, on an exhaustive database offering national coverage. The large study population offered to perform sub-group analyses (by field of surgery, by age group, etc.). Because the surgical procedure and diagnoses are used for costing, the identification of the study population and the cost analysis are an accurate assessment of real-life hospital care of incisional hernia after abdominal surgery. The retrospective data collection and the absence of intervention on the patient to participate in the study prevents selection bias. Patients with more than one abdominal incision were excluded from the risk factor analysis to preclude a confounding bias. The risk factors for hernia repair were independently uncovered by the Cox analysis and the machine learning approach. The strengths of the machine learning analysis are to provide homogeneous groups of patients at high risk of incisional hernia repair and to enable the combination of risk factors (impossible with the Cox model). We iteratively chose patients between those at risk and not at risk by creating subgroups. Additionally, the decision rules can be led by clinicians (such as choosing the first level of the decision tree to be the field of surgery).

The present study, however, has some limitations. First and foremost, this study only examines incisional hernia repairs, i.e., it fails to consider hernias that are not cared for (not inconvenient, too fragile, irreparable, etc.). Besides, our study neglects patients not searching for medical care for incisional hernia but presenting it, as well as those admitted to the hospital with an incisional hernia but discharged without surgical repair. Due to the absence of clinical results in the claims database, even examining doctors’ visits in the community setting (in the existing extension of the PMSI) would not inform on the possible occurrence of an incisional hernia not leading to a hospitalization. Second, due to legal restrictions in the historic analysis of PMSI database (maximum of 9 years prior to current year) and the need to check for medical history during at least 2 years prior to index surgery, we only included patients with an incisional hernia repair within 5 years of the index surgery. Thus, patients presenting an incisional hernia repair beyond 5 years were not captured and this also leads to an underestimation of the hernia recurrence rate. However, three quarters of incisional hernias are diagnosed (but not necessarily repaired) within the first 2 years after initial laparotomy [5]. Third, data from primary care are not available in the PMSI; hence this study does not report incisional hernia care consumption without hospital surgical repair. Hence, this study underestimates the incisional hernia’s public health burden compared to its real life healthcare consumption. Fourth, patients who died before the incisional hernia repair are not included in the study. This introduces a bias for pancreatic cancer patients, who have a high mortality rate. Fifth, it is not possible to totally ascertain the causal relationship between the index surgery and the incisional hernia repair. Yet, to reduce bias and strengthen the potential likelihood of a correlation, we excluded patients with multiple abdominal surgeries. This precaution strengthens the likelihood of a causal relationship between the repaired incisional hernia and the index surgery. Sixth, hepatobiliary and stomach surgeries encompass several different procedures, from cholecystectomy to major hepatectomy and our results may mask differences in risk levels depending on the exact procedure within those wide heterogeneous families (as confirmed by the high risk in patients with laparotomies). Last, the generalisability of the study results could be limited in time because since 2013–2015 —the inclusion period—clinical practice has evolved. Notably, robot-assisted surgery has been continuously expanding. The results of a meta-analysis claimed a decreased risk of incisional hernia after laparoscopy as compared to open surgery, [24] yet the study failed to consider operations with and without the need of a mini-laparotomy for specimen removal. Meanwhile, a nationwide observational study on colon surgery showed that the incidence of incisional hernia did not decrease with mini-invasive approaches [25]. Taken together, these conclusions suggest that our study results could still apply to the majority of abdominal surgeries after the study period.

In addition, some limitations are related to the database. The absence of clinical details on the hernia and the abdominal surgery (such as incision length and surgery duration) prevents the examination of these crucial risk factors [4, 26]. Moreover, lifestyle risk factors and morphometric features are not collected in this claims database (obesity was limited to the collection of the ICD-10 code on the hospital discharge form and was certainly underreported if it had limited impact on the hospital stay). Consequently, the study design was not suitable for studying the impact of known factors (e.g., smoking [7], certain morphometric domains [27], or closure technique of the index surgery), other risk factors, or the physiopathology of incisional hernia. As a study based on a claims database, the quality of the results depends on the quality of the coding of the hernia repair performed at the hospital. In France, hospitals are reimbursed by the NHI for procedures they perform on patients and care they provide. More than 85% of study patients had a medical procedure (CCAM code) of interest and 59% of patients had an ICD-10 code for incisional hernia (47% as PD or RD and 12% as SAD). Overall, the incisional hernia was identified through both a CCAM code and an ICD-10 code in the database in the majority of the study patients, insuring a high level of trust in the validity of the inclusion of the patients.

## Conclusion

In this nationwide population, using an exhaustive healthcare database, at least 4.6% of patients who underwent an abdominal surgical procedure over 2013–2014 were admitted to the hospital for an incisional hernia repair within 5 years, and among them the recurrence rate was 15.6%. The hospital care cost of incisional hernia repair for patients undergoing an abdominal surgery was estimated to be about €4150 per hernia repair.

The field of surgery with the highest risk of subsequent incisional hernia repair was colon and rectum. Also, the machine learning analysis showed that individual risk factors (age starting as early as age 39, or surgery with laparotomy) also put patients operated on other fields of surgery at higher risk of incisional hernia repair. In clinical practice, identifying high-risk patients and applying specific measures and technologies to prevent the onset of incisional hernia is warranted.

## Supplementary Information

Below is the link to the electronic supplementary material.

Supplementary file1 (DOCX 236 KB)

## Data Availability

The data supporting the study findings are part of the SNDS and are available from the HDH (Health Data Hub https://www.health-data-hub.fr/). Restrictions apply to the availability of these data. Permission to access these data was granted by the French data protection authority (Comité National de l’Informatique et des Libertés, CNIL). The study was registered under MR006 with the Health Data Hub on 21/04/2021 (Declaration of conformity No. 2214421v0 of 17/07/2019).

## References

[1] Gignoux B, Bayon Y, Martin D (2021). Incidence and risk factors for incisional hernia and recurrence: retrospective analysis of the French national database. Colorectal Dis.

[2] Muysoms FE, Antoniou SA, Bury K (2015). European hernia society guidelines on the closure of abdominal wall incisions. Hernia.

[3] Dadashzadeh ER, Huckaby LV, Handzel R (2022). The risk of incarceration during nonoperative management of incisional hernias: a population-based analysis of 30,998 patients. Ann Surg.

[4] Söderbäck H, Gunnarsson U, Hellman P, Sandblom G (2018). Incisional hernia after surgery for colorectal cancer: a population-based register study. Int J Colorectal Dis.

[5] Höer J, Lawong G, Klinge U, Schumpelick V (2002). Factors influencing the development of incisional hernia. A retrospective study of 2983 laparotomy patients over a period of 10 years. Chir Z Alle Geb Oper Medizen.

[6] Le Huu NR, Mege D, Ouaïssi M (2012). Incidence and prevention of ventral incisional hernia. J Visc Surg.

[7] Basta MN, Kozak GM, Broach RB (2019). Can we predict incisional hernia?: Development of a surgery-specific decision-support interface. Ann Surg.

[8] Rhemtulla IA, Hsu JY, Broach RB (2021). The incisional hernia epidemic: evaluation of outcomes, recurrence, and expenses using the healthcare cost and utilization project (HCUP) datasets. Hernia J Hernias Abdom Wall Surg.

[9] Bosanquet DC, Ansell J, Abdelrahman T (2015). Systematic review and meta-regression of factors affecting midline incisional hernia rates: analysis of 14,618 patients. PLoS ONE.

[10] van Ramshorst GH, Eker HH, Hop WCJ (2012). Impact of incisional hernia on health-related quality of life and body image: a prospective cohort study. Am J Surg.

[11] Hoffman RD, Danos DM, Lau FH (2021). National health disparities in incisional hernia repair outcomes: an analysis of the healthcare cost and utilization project national inpatient sample (HCUP-NIS) 2012–2014. Surgery.

[12] Rios-Diaz AJ, Morris MP, Christopher AN (2022). National epidemiologic trends (2008–2018) in the United States for the incidence and expenditures associated with incisional hernia in relation to abdominal surgery. Hernia.

[13] Gillion J-F, Sanders D, Miserez M, Muysoms F (2016). The economic burden of incisional ventral hernia repair: a multicentric cost analysis. Hernia.

[14] Itatsu K, Yokoyama Y, Sugawara G (2014). Incidence of and risk factors for incisional hernia after abdominal surgery. Br J Surg.

[15] Goodenough CJ, Ko TC, Kao LS (2015). Development and validation of a risk stratification score for ventral incisional hernia after abdominal surgery: hernia expectation rates in intra-abdominal surgery (The HERNIA Project). J Am Coll Surg.

[16] Köckerling F, Hoffmann H, Adolf D (2021). Potential influencing factors on the outcome in incisional hernia repair: a registry-based multivariable analysis of 22,895 patients. Hernia J Hernias Abdom Wall Surg.

[17] Tuppin P, Rudant J, Constantinou P (2017). Value of a national administrative database to guide public decisions: from the système national d’information interrégimes de l’Assurance Maladie (SNIIRAM) to the système national des données de santé (SNDS) in France. Rev DÉpidémiologie Santé Publique.

[18] Agence Technique de l’Information sur l’Hospitalisation (2021) Etude Nationale des Coûts Médecine Chirurgie Obstétrique - Données 2018. In: ATIH. https://www.atih.sante.fr/enc-mco-donnees-2018. Accessed 5 Apr 2022

[19] Breiman L, Friedman JH, Olshen RA, Stone CJ (2017). Classification and regression trees.

[20] De Oliveira H, Prodel M, Augusto V (2018) Binary Classification on French Hospital Data: Benchmark of 7 Machine Learning Algorithms. In: 2018 IEEE International Conference on Systems, Man, and Cybernetics (SMC). Pp. 1743–1748

[21] Cavailles A, Melloni B, Motola S (2020). Identification of patient profiles with high risk of hospital re-admissions for acute COPD exacerbations (AECOPD) in France using a machine learning model. Int J Chron Obstruct Pulmon Dis.

[22] Bouvier A-M, Launoy G, Bouvier V, et al (2020) Survie des personnes atteintes de cancer en France métropolitaine 1989–2018 - Pancréas. INCa

[23] Holihan JL, Alawadi Z, Martindale RG (2015). Adverse events after ventral hernia repair: the vicious cycle of complications. J Am Coll Surg.

[24] Kössler-Ebs JB, Grummich K, Jensen K (2016). Incisional hernia rates after laparoscopic or open abdominal surgery-a systematic review and meta-analysis. World J Surg.

[25] Jensen KK, Nordholm-Carstensen A, Krarup P-M, Jorgensen LN (2020). Incidence of incisional hernia repair after laparoscopic compared to open resection of colonic cancer: a nationwide analysis of 17,717 patients. World J Surg.

[26] Muysoms FE, Miserez M, Berrevoet F (2009). Classification of primary and incisional abdominal wall hernias. Hernia J Hernias Abdom Wall Surg.

[27] McAuliffe PB, Desai AA, Talwar AA (2022). Preoperative computed tomography morphological features indicative of incisional hernia formation after abdominal surgery. Ann Surg.

