# Supplementary material for: Incidence, risk factors, and burden of incisional hernia repair after abdominal surgery in France: a nationwide study
Source: Hernia. 2023 Jun 27;27(4):861–71. doi: 10.1007/s10029-023-02825-9 (PMC10374769; doi:10.1007/s10029-023-02825-9)

Hernia

**Supplementary Materials**

Incidence, risk factors, and burden of incisional hernia repair after abdominal surgery in France: a nationwide study

[Supplement Methods 1 Attribution of the surgical procedure potentially related to the hernia repair 2](#_Toc129941588)

[Supplement Table 1 CCAM codes for abdominal surgery 3](#_Toc129941589)

[Supplement Table 2 ICD-10 codes considered to capture digestive and urinary stoma 14](#_Toc129941590)

[Supplement Table 3 Codes used to capture comorbidities 15](#_Toc129941591)

[Supplement Table 4 Candidate variables to best discriminate the study population in the machine learning approach 16](#_Toc129941592)

[Supplement Table 5 Multivariable analysis of factors associated with the first incisional hernia repair 17](#_Toc129941593)

[Supplement Figure 1 Number of incisional hernia repairs per patient 19](#_Toc129941594)

[Supplement Figure 2 Factors associated with incisional hernia repair according to the machine learning analyses, 4th level 20](#_Toc129941595)

**Supplementary Methods**

## Supplement Methods 1 Attribution of the surgical procedure potentially related to the hernia repair

During the inclusion hospital stay, if the patient had surgical procedures in multiple fields, the following rules applied to attribute an incisional hernia repair to a single procedure:

1. If the hospital stay included colorectal surgery, this surgery prevailed;
2. If the hospital stay included a laparotomy, this procedure prevailed, irrespective of possible coelioscopies during the same hospital stay;
3. If multiple procedures were performed over several days, the last procedure prevailed;
4. If multiple procedures were performed during the same day, the following order of priority prevailed:
   1. Surgery of the peritoneum,
   2. Stomach surgery,
   3. Pancreas surgery,
   4. Hepatobiliary surgery

**Supplementary Tables**

## Supplement Table 1 CCAM codes for abdominal surgery

CCAM (Classification Commune des Actes Médicaux) codes are the equivalent of the American Current Procedural Terminology (CPT) used for billing purposes.

| **Location** | **CCAM code** | **Label** |
| --- | --- | --- |
| Colon | HHCC001 | Suture de plaie ou de perforation du Colon, par cœlioscopie |
| Colon | HHCA001 | Suture de plaie ou de perforation du Colon, par laparotomie |
| Colon | HHCC007 | Colostomie cutanée, par cœlioscopie |
| Colon | HHCA002 | Colostomie cutanée, par laparotomie |
| Colon | HHMC005 | Réfection de stomie cutanée intestinale, par cœlioscopie |
| Colon | HHMA002 | Réfection de stomie cutanée intestinale, par laparotomie |
| Colon | HHSA001 | Fermeture de colostomie cutanée latérale, par abord direct |
| Colon | HHCC011 | Colocolostomie de dérivation [Anastomose colocolique sans exérèse colique], par cœlioscopie |
| Colon | HHCA003 | Colocolostomie de dérivation [Anastomose colocolique sans exérèse colique], par laparotomie |
| Colon | HHPC002 | Colotomie à visée thérapeutique, par cœlioscopie |
| Colon | HHPA001 | Colotomie à visée thérapeutique, par laparotomie |
| Colon | HHFC296 | Colectomie droite sans rétablissement de la continuité, par cœlioscopie |
| Colon | HHFA026 | Colectomie droite sans rétablissement de la continuité, par laparotomie |
| Colon | HHFA009 | Colectomie droite avec rétablissement de la continuité, par laparotomie |
| Colon | HHFA008 | Colectomie droite avec rétablissement de la continuité, par cœlioscopie ou par laparotomie avec préparation par cœlioscopie |
| Colon | HHFA018 | Colectomie transverse, par laparotomie |
| Colon | HHFA023 | Colectomie transverse, par cœlioscopie ou par laparotomie avec préparation par cœlioscopie |
| Colon | HHFC040 | Colectomie gauche sans libération de l'angle colique gauche, sans  rétablissement de la continuité, par cœlioscopie |
| Colon | HHFA014 | Colectomie gauche sans libération de l'angle colique gauche, sans rétablissement de la continuité, par laparotomie |
| Colon | HHFA017 | Colectomie gauche sans libération de l'angle colique gauche, avec rétablissement de la continuité, par laparotomie |
| Colon | HHFA010 | Colectomie gauche sans libération de l'angle colique gauche, avec rétablissement de la continuité, par cœlioscopie ou par laparotomie avec préparation par cœlioscopie |
| Colon | HHFA024 | Colectomie gauche avec libération de l'angle colique gauche, sans rétablissement de la continuité, par laparotomie |
| Colon | HHFA006 | Colectomie gauche avec libération de l'angle colique gauche, avec rétablissement de la continuité, par laparotomie |
| Colon | HHFA002 | Colectomie gauche avec libération de l'angle colique gauche, avec rétablissement de la continuité, par cœlioscopie ou par laparotomie avec préparation par cœlioscopie |
| Colon | HHFA021 | Colectomie totale avec conservation du rectum, sans rétablissement de la continuité, par laparotomie |
| Colon | HHFA005 | Colectomie totale avec conservation du rectum, sans rétablissement de la continuité, par cœlioscopie ou par laparotomie avec préparation par cœlioscopie |
| Colon | HHFA022 | Colectomie totale avec conservation du rectum, avec anastomose iléorectale, par laparotomie |
| Colon | HHFA004 | Colectomie totale avec conservation du rectum, avec anastomose iléorectale, par cœlioscopie ou par laparotomie avec préparation par cœlioscopie |
| Colon | HHFA030 | Coloproctectomie totale sans rétablissement de la continuité, par laparotomie |
| Colon | HHFA029 | Coloproctectomie totale sans rétablissement de la continuité, par cœlioscopie ou par laparotomie avec préparation par cœlioscopie |
| Colon | HHFA031 | Coloproctectomie totale avec anastomose iléoanale, par laparotomie |
| Colon | HHFA028 | Coloproctectomie totale avec anastomose iléoanale, par cœlioscopie ou par laparotomie avec préparation par cœlioscopie |
| Colon | HHMC001 | Rétablissement secondaire de la continuité digestive après colectomie, par cœlioscopie |
| Colon | HHMA003 | Rétablissement secondaire de la continuité digestive après colectomie, par laparotomie |
| Colon | HHFA003 | Résection du Colon pour malformation congénitale avec rétablissement de la continuité, par laparotomie |
| Diagnostic | ZCQC002 | Exploration de la cavité abdominale, par cœlioscopie [Cœlioscopie exploratrice] |
| Diagnostic | ZCQA001 | Exploration de la cavité abdominale, par laparotomie [Laparotomie exploratrice] |
| Diagnostic | HFPA003 | Gastrotomie exploratrice, par laparotomie |
| Diagnostic | HGPA005 | Duodénotomie exploratrice, par laparotomie |
| Diagnostic | HGPA003 | Entérotomie exploratrice, par laparotomie |
| Diagnostic | HHPC001 | Colotomie exploratrice, par cœlioscopie |
| Diagnostic | HHPA002 | Colotomie exploratrice, par laparotomie |
| Stomach, duodenum | HGCC031 | Suture de plaie ou de perforation de l'intestin grêle, par cœlioscopie |
| Stomach, duodenum | HGCA002 | Suture de plaie ou de perforation de l'intestin grêle, par laparotomie |
| Stomach, duodenum | HGLA001 | Pose d'une sonde de jéjunostomie pour alimentation entérale, par laparotomie |
| Stomach, duodenum | HGCC026 | Entérostomie cutanée, par cœlioscopie |
| Stomach, duodenum | HGCA008 | Entérostomie cutanée, par laparotomie |
| Stomach, duodenum | HGMA001 | Confection secondaire d'une iléostomie continente de Koch, par laparotomie |
| Stomach, duodenum | HGSA001 | Fermeture d'entérostomie cutanée, par abord direct |
| Stomach, duodenum | HGCC003 | Entéroentérostomie de dérivation, par cœlioscopie |
| Stomach, duodenum | HGCA001 | Entéroentérostomie de dérivation, par laparotomie |
| Stomach, duodenum | HGCC015 | Iléocolostomie de dérivation [Anastomose iléocolique sans exérèse intestinale], par cœlioscopie |
| Stomach, duodenum | HGCA005 | Iléocolostomie de dérivation [Anastomose iléocolique sans exérèse intestinale], par laparotomie |
| Stomach, duodenum | HGPC006 | Entérotomie à visée thérapeutique, par cœlioscopie |
| Stomach, duodenum | HGPA002 | Entérotomie à visée thérapeutique, par laparotomie |
| Stomach, duodenum | HGEC009 | Réduction d'invagination intestinale aigüe, par cœlioscopie |
| Stomach, duodenum | HGEA003 | Réduction d'invagination intestinale aigüe, par laparotomie |
| Stomach, duodenum | HGFA001 | Résection de l'angle duodénojéjunal avec rétablissement de la continuité, par laparotomie |
| Stomach, duodenum | HGFA005 | Résection segmentaire unique de l'intestin grêle pour occlusion, par laparotomie |
| Stomach, duodenum | HGFC014 | Résection segmentaire unique de l'intestin grêle sans rétablissement de la continuité, en dehors de l'occlusion, par cœlioscopie |
| Stomach, duodenum | HGFA003 | Résection segmentaire unique de l'intestin grêle sans rétablissement de la continuité, en dehors de l'occlusion, par laparotomie |
| Stomach, duodenum | HGFC021 | Résection segmentaire unique de l'intestin grêle avec rétablissement de la continuité, en dehors de l'occlusion, par cœlioscopie |
| Stomach, duodenum | HGFA007 | Résection segmentaire unique de l'intestin grêle avec rétablissement de la continuité, en dehors de l'occlusion, par laparotomie |
| Stomach, duodenum | HGFC016 | Résection segmentaire multiple de l'intestin grêle, par cœlioscopie |
| Stomach, duodenum | HGFA004 | Résection segmentaire multiple de l'intestin grêle, par laparotomie |
| Stomach, duodenum | HGFA013 | Résection totale de l'intestin grêle, par laparotomie |
| Stomach, duodenum | HGEA005 | Transplantation d'intestin grêle, par laparotomie |
| Stomach, duodenum | HGEA002 | Transplantation d'intestin grêle et de foie réduit, par laparotomie |
| Stomach, duodenum | HGEA004 | Transplantation d'intestin grêle et de foie total, par laparotomie |
| Stomach, duodenum | HGFC002 | Résection du diverticule iléal [de Meckel], par cœlioscopie |
| Stomach, duodenum | HGFA006 | Résection du diverticule iléal [de Meckel], par laparotomie |
| Stomach, duodenum | HGAA002 | Plastie d'allongement de l'intestin grêle, par laparotomie |
| Stomach, duodenum | HGEC001 | Détorsion intestinale ou section de bride péritonéale pour vice de rotation de l'anse intestinale primitive [mesenterium commune], par cœlioscopie |
| Stomach, duodenum | HGEA001 | Détorsion intestinale ou section de bride péritonéale pour vice de rotation de l'anse intestinale primitive [mesenterium commune], par laparotomie |
| Stomach, duodenum | HGAC010 | Plastie d'élargissement unique ou multiple de l'intestin grêle, par cœlioscopie |
| Stomach, duodenum | HGAA003 | Plastie d'élargissement unique ou multiple de l'intestin grêle, par laparotomie |
| Stomach, duodenum | HGMA005 | Rétablissement secondaire de la continuité digestive après résection de l'intestin grêle, par laparotomie |
| Stomach, duodenum | HHFA016 | Appendicectomie, par cœlioscopie ou par laparotomie avec préparation par cœlioscopie |
| Stomach, duodenum | HHFA011 | Appendicectomie, par laparotomie |
| Stomach, duodenum | HHFA001 | Appendicectomie, par abord de la fosse iliaque |
| Stomach, duodenum | HHFA025 | Appendicectomie avec toilette péritonéale pour péritonite aigüe généralisée, par cœlioscopie ou par laparotomie avec préparation par cœlioscopie |
| Stomach, duodenum | HHFA020 | Appendicectomie avec toilette péritonéale pour péritonite aigüe généralisée, par laparotomie |
| Stomach, duodenum | HFCC001 | Suture de plaie ou de perforation de l'estomac ou du duodénum, par cœlioscopie |
| Stomach, duodenum | HFCA003 | Suture de plaie ou de perforation de l'estomac ou du duodénum, par laparotomie |
| Stomach, duodenum | HFCC002 | Gastrostomie cutanée, par cœlioscopie |
| Stomach, duodenum | HFCA002 | Gastrostomie cutanée, par laparotomie |
| Stomach, duodenum | HFSA001 | Fermeture de gastrostomie cutanée, par abord direct |
| Stomach, duodenum | HFMC004 | Confection d'une valve tubérositaire sans libération de la grande courbure gastrique, par cœlioscopie |
| Stomach, duodenum | HFMA003 | Confection d'une valve tubérositaire sans libération de la grande courbure gastrique, par laparotomie |
| Stomach, duodenum | HFMC001 | Confection d'une valve tubérositaire avec libération de la grande courbure gastrique, par cœlioscopie |
| Stomach, duodenum | HFMA008 | Confection d'une valve tubérositaire avec libération de la grande courbure gastrique, par laparotomie |
| Stomach, duodenum | HFCC022 | Gastrojéjunostomie de dérivation [Gastro-entéro-anastomose sans résection gastrique], par cœlioscopie |
| Stomach, duodenum | HFCA004 | Gastrojéjunostomie de dérivation [Gastro-entéro-anastomose sans résection gastrique], par laparotomie |
| Stomach, duodenum | HGCA006 | Dérivation des sécrétions duodénales et biliopancréatiques par réfection du montage après gastrectomie [Diversion du duodénum], par laparotomie |
| Stomach, duodenum | HFPA001 | Gastrotomie à visée thérapeutique, par laparotomie |
| Stomach, duodenum | HGPA001 | Duodénotomie à visée thérapeutique ou duodénectomie partielle, par laparotomie |
| Stomach, duodenum | HFMC003 | Pyloroplastie ou duodénoplastie, par cœlioscopie |
| Stomach, duodenum | HFMA004 | Pyloroplastie ou duodénoplastie, par laparotomie |
| Stomach, duodenum | HFLC900 | Implantation souscutanée d'un stimulateur gastrique avec pose d'une sonde pariétale gastrique pour obésité morbide, par cœlioscopie |
| Stomach, duodenum | HFGC900 | Ablation d'une sonde de stimulation pariétale gastrique pour obésité morbide, par cœlioscopie |
| Stomach, duodenum | HGCC027 | Court-circuit biliopancréatique ou intestinal pour obésité morbide, par cœlioscopie |
| Stomach, duodenum | HGCA009 | Court-circuit biliopancréatique ou intestinal pour obésité morbide, par laparotomie |
| Stomach, duodenum | HFCC003 | Court-circuit [Bypass] gastrique pour obésité morbide, par cœlioscopie |
| Stomach, duodenum | HFCA001 | Court-circuit [Bypass] gastrique pour obésité morbide, par laparotomie |
| Stomach, duodenum | HFFC018 | Gastrectomie longitudinale [Sleeve gastrectomy] pour obésité morbide, par cœlioscopie |
| Stomach, duodenum | HFFA011 | Gastrectomie longitudinale [Sleeve gastrectomy] pour obésité morbide, par laparotomie |
| Stomach, duodenum | HFFC004 | Gastrectomie avec court-circuit biliopancréatique ou intestinal pour obésité morbide, par cœlioscopie |
| Stomach, duodenum | HFFA001 | Gastrectomie avec court-circuit biliopancréatique ou intestinal pour obésité morbide, par laparotomie |
| Stomach, duodenum | HFMC006 | Gastroplastie verticale calibrée pour obésité morbide, par cœlioscopie |
| Stomach, duodenum | HFMA010 | Gastroplastie verticale calibrée pour obésité morbide, par laparotomie |
| Stomach, duodenum | HFMC007 | Gastroplastie par pose d'anneau ajustable périgastrique pour obésité morbide, par cœlioscopie |
| Stomach, duodenum | HFMA009 | Gastroplastie par pose d'anneau ajustable périgastrique pour obésité morbide, par laparotomie |
| Stomach, duodenum | HFKC001 | Changement d'un anneau ajustable périgastrique pour obésité morbide, par cœlioscopie |
| Stomach, duodenum | HFKA002 | Changement d'un anneau ajustable périgastrique pour obésité morbide, par laparotomie |
| Stomach, duodenum | HFMC008 | Repositionnement ou ablation d'un anneau ajustable périgastrique, par cœlioscopie |
| Stomach, duodenum | HFMA011 | Repositionnement ou ablation d'un anneau ajustable périgastrique, par laparotomie |
| Stomach, duodenum | HFKA001 | Changement ou repositionnement du dispositif d'accès d'un anneau ajustable périgastrique pour obésité morbide, par abord direct |
| Stomach, duodenum | HFDC002 | Gastropexie postérieure [Cardiopexie], par cœlioscopie |
| Stomach, duodenum | HFDA002 | Gastropexie postérieure [Cardiopexie], par laparotomie |
| Stomach, duodenum | HFFC001 | Résection partielle atypique de la paroi de l'estomac n'interrompant pas la continuité, par cœlioscopie |
| Stomach, duodenum | HFFA009 | Résection partielle atypique de la paroi de l'estomac n'interrompant pas la continuité, par laparotomie |
| Stomach, duodenum | HFFA003 | Gastrectomie partielle supérieure [polaire supérieure] avec rétablissement de la continuité, par laparotomie |
| Stomach, duodenum | HFFC012 | Gastrectomie partielle inférieure avec anastomose gastroduodénale, par cœlioscopie |
| Stomach, duodenum | HFFA002 | Gastrectomie partielle inférieure avec anastomose gastroduodénale, par laparotomie |
| Stomach, duodenum | HFFC002 | Gastrectomie partielle inférieure avec anastomose gastrojéjunale, par cœlioscopie |
| Stomach, duodenum | HFFA006 | Gastrectomie partielle inférieure avec anastomose gastrojéjunale, par laparotomie |
| Stomach, duodenum | HFFC017 | Gastrectomie totale avec rétablissement de la continuité, par cœlioscopie |
| Stomach, duodenum | HFFA005 | Gastrectomie totale avec rétablissement de la continuité, par laparotomie |
| Stomach, duodenum | HFFA008 | Dégastrogastrectomie partielle avec rétablissement de la continuité, par laparotomie |
| Stomach, duodenum | HFMA005 | Totalisation secondaire de gastrectomie avec rétablissement de la continuité, par laparotomie |
| Stomach, duodenum | HFPC001 | Pylorotomie extramuqueuse [Pyloromyotomie extramuqueuse], par cœlioscopie |
| Stomach, duodenum | HFPA002 | Pylorotomie extramuqueuse [Pyloromyotomie extramuqueuse], par laparotomie |
| Stomach, duodenum | HGFA014 | Exérèse de la papille duodénale majeure, par laparotomie |
| Stomach, duodenum | HGCA007 | Exclusion du duodénum, par laparotomie |
| Stomach, duodenum | HGMA002 | Remise en circuit secondaire du duodénum, par laparotomie |
| Hepatobiliary | HMCA009 | Cholécystostomie cutanée, par laparotomie |
| Hepatobiliary | HMLC001 | Pose d'endoprothèse biliaire par cholédochotomie, par cœlioscopie |
| Hepatobiliary | HMLA001 | Pose d'endoprothèse biliaire par cholédochotomie, par laparotomie |
| Hepatobiliary | HLJC001 | Évacuation de collection hépatique, par cœlioscopie |
| Hepatobiliary | HLJA001 | Évacuation de collection hépatique, par laparotomie |
| Hepatobiliary | HLCC001 | Fenestration de kystes biliaires hépatiques, par cœlioscopie |
| Hepatobiliary | HLCA002 | Fenestration de kystes biliaires hépatiques, par laparotomie |
| Hepatobiliary | HMCC003 | Cholécystogastrostomie ou cholécystoduodénostomie, par cœlioscopie |
| Hepatobiliary | HMCA003 | Cholécystogastrostomie ou cholécystoduodénostomie, par laparotomie |
| Hepatobiliary | HMCA010 | Cholécystogastrostomie ou cholécystoduodénostomie avec gastrojéjunostomie, par laparotomie |
| Hepatobiliary | HMCA001 | Cholécystoduodénostomie pour atrésie des conduits biliaires extrahépatiques, par laparotomie |
| Hepatobiliary | HMCA011 | Cholécystojéjunostomie, par laparotomie |
| Hepatobiliary | HMCC002 | Cholédochoduodénostomie, par cœlioscopie |
| Hepatobiliary | HMCA002 | Cholédochoduodénostomie, par laparotomie |
| Hepatobiliary | HMCC013 | Cholédochoduodénostomie avec gastrojéjunostomie, par cœlioscopie |
| Hepatobiliary | HMCA004 | Cholédochoduodénostomie avec gastrojéjunostomie, par laparotomie |
| Hepatobiliary | HMCC001 | Cholédochojéjunostomie, par cœlioscopie |
| Hepatobiliary | HMCA006 | Cholédochojéjunostomie, par laparotomie |
| Hepatobiliary | HMCA008 | Anastomose biliodigestive portant sur la convergence des conduits hépatiques, par laparotomie |
| Hepatobiliary | HMCA007 | Anastomose biliodigestive au-dessus de la convergence portant sur plusieurs conduits biliaires, par laparotomie |
| Hepatobiliary | HMCA005 | Anastomose biliodigestive intrahépatique portant sur un conduit biliaire segmentaire, par laparotomie |
| Hepatobiliary | HMGC001 | Ablation de calcul de la voie biliaire principale par cholédochotomie, par cœlioscopie |
| Hepatobiliary | HMGA001 | Ablation de calcul de la voie biliaire principale par cholédochotomie, par laparotomie |
| Hepatobiliary | HMFC004 | Cholécystectomie, par cœlioscopie |
| Hepatobiliary | HMFA007 | Cholécystectomie, par laparotomie |
| Hepatobiliary | HMFC001 | Cholécystectomie avec ablation transcystique de calcul de la voie biliaire principale, par cœlioscopie |
| Hepatobiliary | HMFA004 | Cholécystectomie avec ablation transcystique de calcul de la voie biliaire principale, par laparotomie |
| Hepatobiliary | HMFC002 | Cholécystectomie avec ablation de calcul de la voie biliaire principale par cholédochotomie, par cœlioscopie |
| Hepatobiliary | HMFA003 | Cholécystectomie par cœlioscopie, avec ablation de calcul de la voie biliaire principale par cholédochotomie, par laparotomie |
| Hepatobiliary | HMFA008 | Cholécystectomie avec ablation de calcul de la voie biliaire principale par cholédochotomie, par laparotomie |
| Hepatobiliary | HMFC003 | Cholécystectomie avec cholédochogastrostomie ou cholédochoduodénostomie, par cœlioscopie |
| Hepatobiliary | HMFA006 | Cholécystectomie par cœlioscopie, avec cholédochoduodénostomie par laparotomie |
| Hepatobiliary | HMFA002 | Cholécystectomie avec cholédochogastrostomie ou cholédochoduodénostomie, par laparotomie |
| Hepatobiliary | HMFC005 | Cholécystectomie avec cholédochojéjunostomie, par cœlioscopie |
| Hepatobiliary | HMFA005 | Cholécystectomie par cœlioscopie, avec cholédochojéjunostomie par laparotomie |
| Hepatobiliary | HMFA001 | Cholécystectomie avec cholédochojéjunostomie, par laparotomie |
| Hepatobiliary | HMFA009 | Résection de la voie biliaire principale pédiculaire avec anastomose biliodigestive, par laparotomie |
| Hepatobiliary | HMFA010 | Résection de la voie biliaire principale pédiculaire et intrapancréatique avec anastomose biliodigestive, par laparotomie |
| Hepatobiliary | HLNA007 | Destruction de tumeur hépatique avec courant de radiofréquence, par laparotomie |
| Hepatobiliary | HLFA014 | Séquestrectomie hépatique, par laparotomie |
| Hepatobiliary | HLFA012 | Kystectomie ou périkystectomie hépatique, par laparotomie |
| Hepatobiliary | HLFA002 | Résection du dôme saillant de kyste hydatique du foie, par laparotomie |
| Hepatobiliary | HLFC003 | Résection atypique du foie, par cœlioscopie |
| Hepatobiliary | HLFA019 | Résection atypique du foie, par laparotomie |
| Hepatobiliary | HLFC004 | Unisegmentectomie hépatique, par cœlioscopie |
| Hepatobiliary | HLFA020 | Unisegmentectomie hépatique, par laparotomie |
| Hepatobiliary | HLFA003 | Résection du lobe caudé [de Spigel] [segment I] du foie, par laparotomie |
| Hepatobiliary | HLFC027 | Bisegmentectomie hépatique, par cœlioscopie |
| Hepatobiliary | HLFA009 | Bisegmentectomie hépatique, par laparotomie |
| Hepatobiliary | HLFC032 | Trisegmentectomie hépatique, par cœlioscopie |
| Hepatobiliary | HLFA006 | Trisegmentectomie hépatique, par laparotomie |
| Hepatobiliary | HLFC002 | Lobectomie hépatique gauche, par cœlioscopie |
| Hepatobiliary | HLFA011 | Lobectomie hépatique gauche, par laparotomie |
| Hepatobiliary | HLFC037 | Hépatectomie gauche, par cœlioscopie |
| Hepatobiliary | HLFA018 | Hépatectomie gauche, par laparotomie |
| Hepatobiliary | HLFA007 | Hépatectomie gauche élargie au lobe caudé [de Spigel] [segment I], par laparotomie |
| Hepatobiliary | HLFA017 | Hépatectomie droite, par laparotomie |
| Hepatobiliary | HLFA004 | Hépatectomie droite élargie au lobe caudé [de Spigel] [segment I], par laparotomie |
| Hepatobiliary | HLFA005 | Lobectomie hépatique droite [Hépatectomie droite élargie au segment IV], par laparotomie |
| Hepatobiliary | HLFA010 | Hépatectomie centrale, par laparotomie |
| Hepatobiliary | HLFA015 | Prélèvement d'un greffon hépatique, chez un sujet vivant |
| Hepatobiliary | HLEA002 | Transplantation de foie réduit |
| Hepatobiliary | HLEA001 | Transplantation de foie total |
| Hepatobiliary | HLSC012 | Hémostase de lésion du foie, par cœlioscopie |
| Hepatobiliary | HLSA001 | Hémostase de lésion du foie, par laparotomie |
| Pancreas | HNJC001 | Drainage externe de collection pancréatique, par cœlioscopie |
| Pancreas | HNJA001 | Drainage externe de collection pancréatique, par laparotomie |
| Pancreas | HNCC021 | Anastomose entre un faux kyste du Pancreas et l'estomac [Kystogastrostomie], par cœlioscopie |
| Pancreas | HNCA008 | Anastomose entre un faux kyste du Pancreas et l'estomac [Kystogastrostomie], par laparotomie |
| Pancreas | HNCA001 | Anastomose entre un faux kyste du Pancreas et le duodénum [Kystoduodénostomie], par laparotomie |
| Pancreas | HNCC033 | Anastomose entre un faux kyste du Pancreas et le jéjunum [Kystojéjunostomie], par cœlioscopie |
| Pancreas | HNCA005 | Anastomose entre un faux kyste du Pancreas et le jéjunum [Kystojéjunostomie], par laparotomie |
| Pancreas | HNCA006 | Suture de plaie du Pancreas avec reconstruction du conduit pancréatique, par laparotomie |
| Pancreas | HNCA007 | Anastomose pancréaticojéjunale, par laparotomie |
| Pancreas | HNCA002 | Anastomose pancréaticojéjunale avec anastomose biliojéjunale, par laparotomie |
| Pancreas | HNCA004 | Anastomose pancréaticojéjunale avec gastrojéjunostomie, par laparotomie |
| Pancreas | HNCA003 | Anastomose pancréaticojéjunale avec anastomose biliojéjunale et gastrojéjunostomie, par laparotomie |
| Pancreas | HNFC027 | Nécrosectomie pancréatique, par cœlioscopie |
| Pancreas | HNFA012 | Nécrosectomie pancréatique, par laparotomie |
| Pancreas | HNFC001 | Exérèse de tumeur du Pancreas, par cœlioscopie |
| Pancreas | HNFA005 | Exérèse de tumeur du Pancreas, par laparotomie |
| Pancreas | HNFC028 | Pancréatectomie gauche avec conservation de la rate, par cœlioscopie |
| Pancreas | HNFA008 | Pancréatectomie gauche avec conservation de la rate, par laparotomie |
| Pancreas | HNFA002 | Pancréatectomie gauche avec conservation de la rate, avec anastomose pancréatojéjunale ou pancréaticojéjunale, par laparotomie |
| Pancreas | HNFC002 | Pancréatectomie gauche avec splénectomie [Splénopancréatectomie gauche], par cœlioscopie |
| Pancreas | HNFA013 | Pancréatectomie gauche avec splénectomie [Splénopancréatectomie gauche], par laparotomie |
| Pancreas | HNFA010 | Pancréatectomie gauche avec splénectomie [Splénopancréatectomie gauche] avec anastomose pancréatojéjunale ou pancréaticojéjunale, par laparotomie |
| Pancreas | HNFA001 | Isthmectomie pancréatique avec rétablissement de continuité du conduit pancréatique, par laparotomie |
| Pancreas | HNFA011 | Pancréatectomie totale ou subtotale avec conservation du duodénum, sans splénectomie, par laparotomie |
| Pancreas | HNFA006 | Pancréatectomie totale ou subtotale avec conservation du duodénum et splénectomie, par laparotomie |
| Pancreas | HNFA007 | Duodénopancréatectomie céphalique, par laparotomie |
| Pancreas | HNFA004 | Duodénopancréatectomie totale avec splénectomie [Splénopancréatectomie totale], par laparotomie |
| Pancreas | HNEA900 | Transplantation du Pancreas, par laparotomie |
| Pancreas | HNEA002 | Transplantation du Pancreas et du rein, par laparotomie |
| Other procedures on the small bowel or the peritoneum | HPLB003 | Administration intrapéritonéale d'agent pharmacologique anticancéreux avec hyperthermie [Chimiohyperthermie anticancéreuse intrapéritonéale], sous anesthésie générale |
| Other procedures on the small bowel or the peritoneum | ZCJA002 | Évacuation d'une collection intraabdominale, par laparotomie |
| Other procedures on the small bowel or the peritoneum | ZCJA004 | Évacuation de plusieurs collections intraabdominales, par laparotomie |
| Other procedures on the small bowel or the peritoneum | HPCA001 | Dérivation péritonéoveineuse |
| Other procedures on the small bowel or the peritoneum | HPPC003 | Section de bride et/ou d'adhérences péritonéales pour occlusion intestinale aigüe, par cœlioscopie |
| Other procedures on the small bowel or the peritoneum | HPPA002 | Section de bride et/ou d'adhérences péritonéales pour occlusion intestinale aigüe, par laparotomie |
| Other procedures on the small bowel or the peritoneum | HGPC015 | Libération étendue de l'intestin grêle [Entérolyse étendue] pour occlusion aigüe, par cœlioscopie |
| Other procedures on the small bowel or the peritoneum | HGPA004 | Libération étendue de l'intestin grêle [Entérolyse étendue] pour occlusion aigüe, par laparotomie |
| Other procedures on the small bowel or the peritoneum | HPNC001 | Destruction et/ou exérèse de lésion endométriosique superficielle du péritoine, par cœlioscopie |
| Other procedures on the small bowel or the peritoneum | HPNA001 | Destruction et/ou exérèse de lésion endométriosique superficielle du péritoine, par laparotomie |
| Other procedures on the small bowel or the peritoneum | HPFC001 | Exérèse de lésion d'un repli péritonéal [méso] sans résection intestinale, par cœlioscopie |
| Other procedures on the small bowel or the peritoneum | HPFA003 | Exérèse de lésion d'un repli péritonéal [méso] sans résection intestinale, par laparotomie |
| Other procedures on the small bowel or the peritoneum | HPFC002 | Résection du grand omentum [grand épiploon] [Omentectomie], par cœlioscopie |
| Other procedures on the small bowel or the peritoneum | HPFA004 | Résection du grand omentum [grand épiploon] [Omentectomie], par laparotomie |
| Other procedures on the small bowel or the peritoneum | HPBA001 | Réduction de volume tumoral d'une maladie gélatineuse du péritoine, par laparotomie |

## Supplement Table 2 ICD-10 codes considered to capture digestive and urinary stoma

| **ICD-10 code** | **Label** |
| --- | --- |
| Z931 | Gastrostomy status |
| Z932 | Ileostomy status |
| Z933 | Colostomy status |
| Z934 | Other artificial openings of gastrointestinal tract status |
| Z935 | Cystostomy status |
| Z936 | Other artificial openings of urinary tract status |

## Supplement Table 3 Codes used to capture comorbidities

| **Comorbidity** | **Codes** |
| --- | --- |
| Obesity | ICD-10: E66 |
| Cancer | ICD-10: C, D00–D09 |
| Heart failure | ICD-10: I50, I110, I130, I132, I139 |
| Diabetes | ICD-10: E10-E14 |
| Chronic Obstructive Pulmonary Disease | ICD-10: J40-J47, J96 [excluding J960 et J969], J98 |
| Cirrhosis of liver | ICD-10: K74 |
| Chronic kidney disease | ICD-10: N18  DRG: 11K021, 11K022, 11K023, 11K024, 11K02J, 28Z01Z, 28Z02Z, 28Z03Z, 28Z24Z  CCAM: JVJB001, JVJF004, JVJF008, JVRP004, JVRP007, JVRP008, YYYY007 |

CCAM (Classification Commune des Actes Médicaux) codes are the equivalent of the American Current Procedural Terminology (CPT) used for billing purposes; DRG: disease-related group.

## Supplement Table 4 Candidate variables to best discriminate the study population in the machine learning approach

| Sex |
| --- |
| Age |
| Type of surgery at inclusion |
| Type of hospital for the index surgery |
| Number of procedures in the hospital of the index surgery |
| Referral from emergency room |
| Coelioscopy at index surgery |
| Laparotomy at index surgery |
| Obesity |
| Cancer |
| Heart failure |
| Diabetes |
| Cirrhosis |
| Chronic obstructive pulmonary disease |
| Renal failure |

**Supplementary Figures**

## Supplement Table 5 Multivariable analysis of factors associated with the first incisional hernia repair

| Variable | Value | Patients with incisional hernia repair n=22,944 | Total number of patients n=672,429 | Hazard ratio | 95% Confidence interval | P-value (for the value) | P-value (for the variable) |
| --- | --- | --- | --- | --- | --- | --- | --- |
| Sex | Female | 12,886 | 419,495 | 1.000 |  | . | <.0001 |
|  | Male | 10,058 | 252,934 | 1.066 | [1.037;1.095] | <.0001 |  |
| Age (years) | 18-35 | 1,571 | 153,302 | 1.000 |  | . | <.0001 |
|  | 36-55 | 6,790 | 203,280 | 2.958 | [2.798;3.127] | <.0001 |  |
|  | 56-70 | 8,875 | 168,550 | 4.060 | [3.837;4.297] | <.0001 |  |
|  | 71+ | 5,708 | 147,297 | 2.791 | [2.626;2.966] | <.0001 |  |
| Index hospital stay duration (days) | 0-2 | 2,664 | 190,787 | 1.000 |  | . | <.0001 |
|  | 3-4 | 3,171 | 157,254 | 1.225 | [1.162;1.291] | <.0001 |  |
|  | 5-9 | 7,482 | 175,659 | 1.992 | [1.898;2.090] | <.0001 |  |
|  | 10+ | 9,627 | 148,729 | 3.098 | [2.940;3.265] | <.0001 |  |
| Field of surgery (index hospital stay) | Hepatobiliary | 5,245 | 244,694 | 1.000 |  | . | <.0001 |
|  | Stomach, duodenum | 3,351 | 105,377 | 1.035 | [0.985;1.087] | 0.1708 |  |
|  | Pancreas | 454 | 7,090 | 1.097 | [0.991;1.215] | 0.0739 |  |
|  | Colon | 10,293 | 217,602 | 1.233 | [1.180;1.289] | <.0001 |  |
|  | Other procedures on the small bowel or the peritoneum | 3,601 | 97,666 | 1.377 | [1.316;1.442] | <.0001 |  |
| Hospital status (index hospital stay) | Private | 10,090 | 295,629 | 1.000 |  | . | <.0001 |
|  | Public | 12,854 | 376,800 | 0.882 | [0.857;0.907] | <.0001 |  |
| Volume of activity of the hospital (index hospital stay) | 1^st^ quartile | 2,751 | 85,895 | 1.000 |  | . | 0.0133 |
|  | 2^nd^ quartile | 8,611 | 258,602 | 1.000 | [0.958;1.044] | 0.9991 |  |
|  | 3^rd^ quartile | 6,381 | 187,413 | 1.021 | [0.976;1.068] | 0.3618 |  |
|  | 4^th^ quartile | 5,201 | 140,519 | 1.059 | [1.010;1.111] | 0.0186 |  |
| Referral from emergency room (index hospital stay) | No | 17,774 | 504,087 | 1.000 |  | . | <.0001 |
|  | Yes | 5,170 | 168,342 | 0.793 | [0.766;0.821] | <.0001 |  |
| Coelioscopy | No | 14,838 | 295,621 | 1.000 |  | . | 0.9867 |
|  | Yes | 8,106 | 376,808 | 1.001 | [0.889;1.127] | 0.9867 |  |
| Laparotomy | No | 7,826 | 372,306 | 1.000 |  | . | <.0001 |
|  | Yes | 15,118 | 300,123 | 1.877 | [1.661;2.122] | <.0001 |  |
| Obesity | No | 16,202 | 523,911 | 1.000 |  | . | <.0001 |
|  | Yes | 6,742 | 148,518 | 1.875 | [1.813;1.938] | <.0001 |  |
| Cancer | No | 15,984 | 541,610 | 1.000 |  | . | <.0001 |
|  | Yes | 6,960 | 130,819 | 0.912 | [0.881;0.943] | <.0001 |  |
| Heart failure | No | 22,256 | 652,550 | 1.000 |  | . | <.0001 |
|  | Yes | 688 | 19,879 | 0.749 | [0.692;0.811] | <.0001 |  |
| Diabetes | No | 19,715 | 605,570 | 1.000 |  | . | 0.0118 |
|  | Yes | 3,229 | 66,859 | 0.951 | [0.914;0.989] | 0.0118 |  |
| Cirrhosis | No | 22,640 | 667,339 | 1.000 |  | . | 0.0288 |
|  | Yes | 304 | 5,090 | 1.138 | [1.014;1.279] | 0.0288 |  |
| COPD | No | 20,508 | 628,395 | 1.000 |  | . | <.0001 |
|  | Yes | 2,436 | 44,034 | 1.270 | [1.216;1.326] | <.0001 |  |
| Chronic kidney disease | No | 22,151 | 653,702 | 1.000 |  | . | 0.0948 |
|  | Yes | 793 | 18,727 | 0.939 | [0.873;1.011] | 0.0948 |  |

COPD: chronic obstructive pulmonary disease

## Supplement Figure 1 Number of incisional hernia repairs per patient


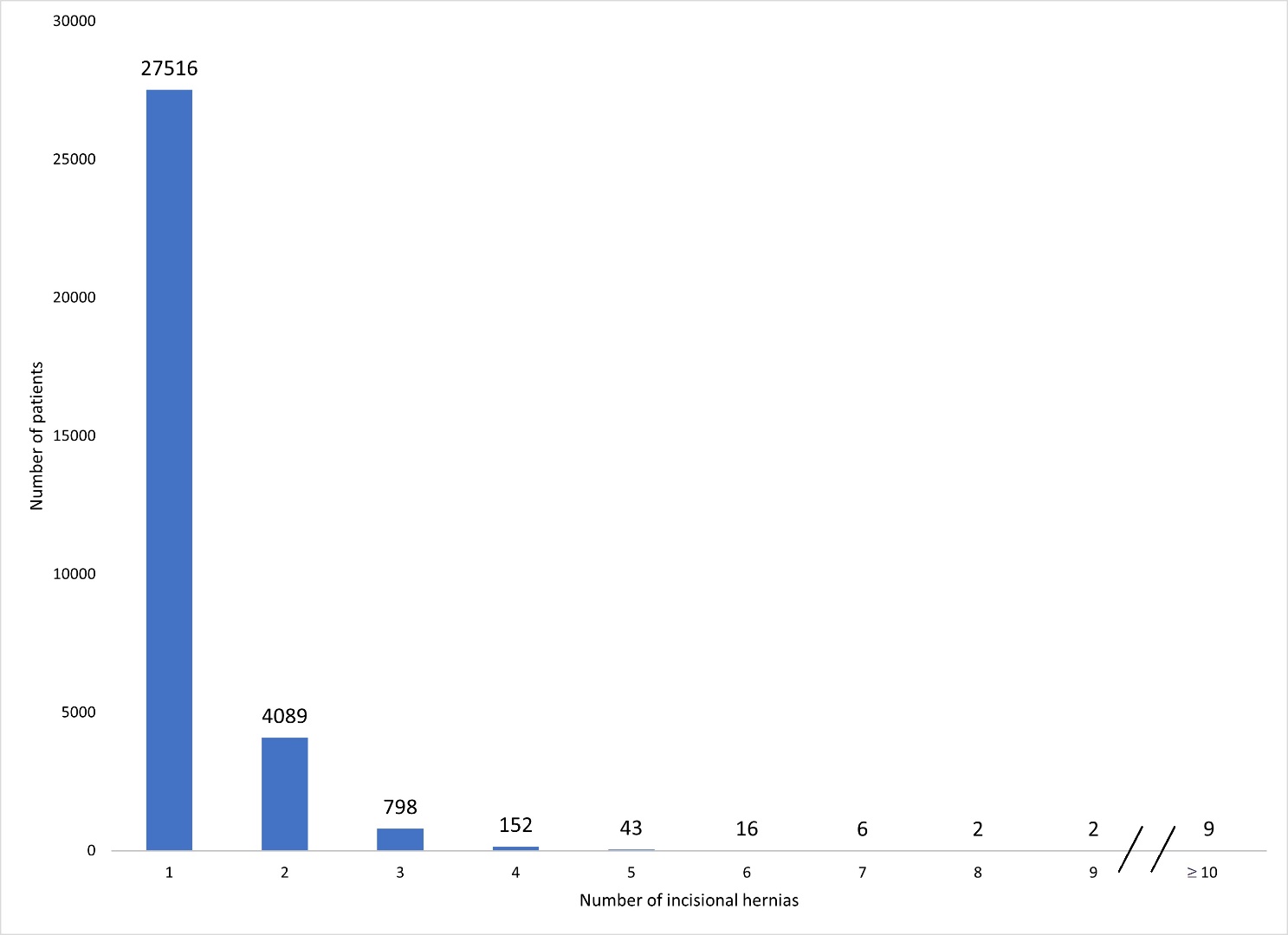


## Supplement Figure 2 Factors associated with incisional hernia repair according to the machine learning analyses, 4th level


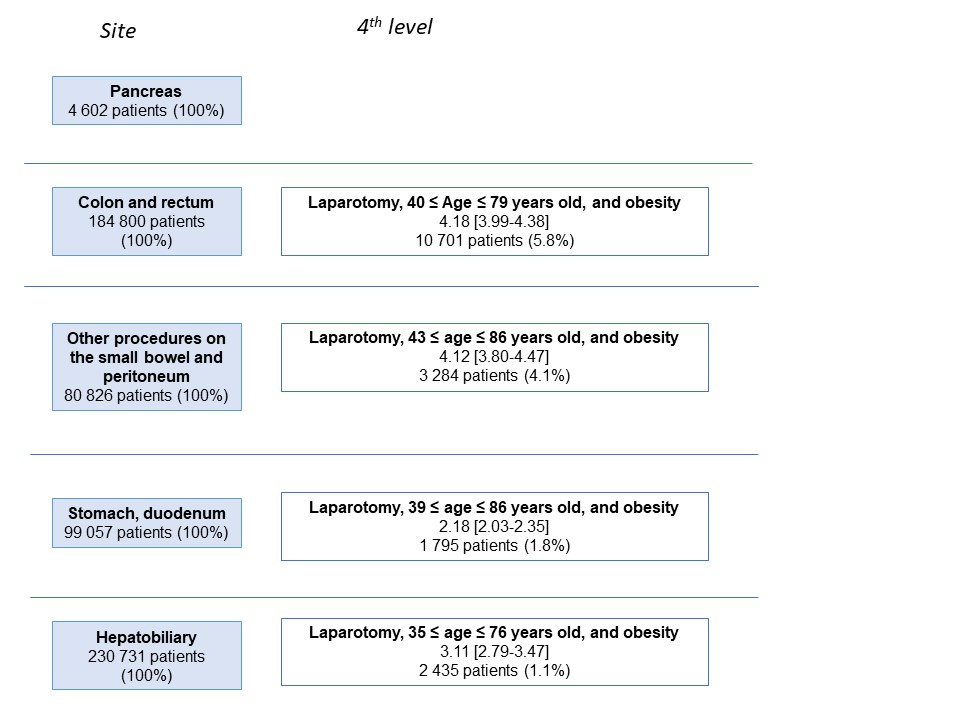

Supplement: Supplementary file 1 — Supplementary file1 (DOCX 236 KB) [file 10029_2023_2825_MOESM1_ESM.docx]
